# Prediction of Radiation Pneumonitis Using Genome-Scale Flux Analysis of RNA-Seq Derived From Peripheral Blood

**DOI:** 10.3389/fmed.2021.715961

**Published:** 2021-08-31

**Authors:** Siqi Yang, Yi Yao, Yi Dong, Junqi Liu, Yingge Li, Lina Yi, Yani Huang, Yanjun Gao, Junping Yin, Qingqing Li, Dafu Ye, Hongyun Gong, Bin Xu, Jian Li, Qibin Song

**Affiliations:** ^1^Cancer Center, Renmin Hospital of Wuhan University, Wuhan, China; ^2^Hubei Provincial Research Center for Precision Medicine of Cancer, Wuhan, China; ^3^Department of Radiation Oncology, The First Affiliated Hospital of Zhengzhou University, Zhengzhou, China; ^4^Oncology Department, Zhongxiang Hospital, Renmin Hospital of Wuhan University, Zhongxiang, China; ^5^Institute of Experimental Immunology, University Clinic of Rheinische Friedrich-Wilhelms-University, Bonn, Germany

**Keywords:** radiation pneumonitis, prediction model, peripheral blood, RNA-Seq, chest radiotherapy, risk prediction, transcriptome analysis

## Abstract

**Purpose:** Radiation pneumonitis (RP) frequently occurs during a treatment course of chest radiotherapy, which significantly reduces the clinical outcome and efficacy of radiotherapy. The ability to easily predict RP before radiotherapy would allow this disease to be avoided.

**Methods and Materials:** This study recruited 48 lung cancer patients requiring chest radiotherapy. For each participant, RNA sequencing (RNA-Seq) was performed on a peripheral blood sample before radiotherapy. The RNA-Seq data was then integrated into a genome-scale flux analysis to develop an RP scoring system for predicting the probability of occurrence of RP. Meanwhile, the clinical information and radiation dosimetric parameters of this cohort were collected for analysis of any statistical associations between these parameters and RP. A non-parametric rank sum test showed no significant difference between the predicted results from the RP score system and the clinically observed occurrence of RP in this cohort.

**Results:** The results of the univariant analysis suggested that the tumor stage, exposure dose, and bilateral lung dose of V5 and V20 were significantly associated with the occurrence of RP. The results of the multivariant analysis suggested that the exposure doses of V5 and V20 were independent risk factors associated with RP and a level of RP ≥ 2, respectively. Thus, our results indicate that our RP scoring system could be applied to accurately predict the risk of RP before radiotherapy because the scores were highly consistent with the clinically observed occurrence of RP.

**Conclusion:** Compared with the standard statistical methods, this genome-scale flux-based scoring system is more accurate, straightforward, and economical, and could therefore be of great significance when making clinical decisions for chest radiotherapy.

## Introduction

Radiation pneumonitis (RP) is a common treatment complication in cancer patients who receive chest radiotherapy, usually occurring within 6 weeks to 3 months of the end of radiation therapy (1). Severe RP can even lead to death (2). Therefore, RP not only limits the implementation of radiotherapy, but also significantly reduces the treatment efficacy and quality of life of cancer patients. To date, a variety of risk factors related to RP have been reported in diverse clinical studies, such as the radiation dose, the exposure lung volume, the average lung exposure dose, sex, age, chemotherapy, and smoking history (3). Although those risk factors contain relevant information related to RP, prediction models based on those risk factors alone have demonstrated limited performance with low degrees of sensitivity and specificity for predicting the occurrence of RP.

From a pathophysiological perspective, the alveolar epithelial and pulmonary interstitial cells are injured during or after the exposure of lung tissue to ionizing radiation. These injured cells continuously release a variety of inflammatory cytokines that induce inflammation in local lung tissue (4). The released inflammatory cytokines accumulate, causing severe damage to lung tissue, and enter the blood circulation, which indirectly induces systemic inflammatory reactions (5).

Messenger RNA (mRNA) is an important part of the synthesis of cytokines and therefore plays an essential role in the occurrence and development of RP (6). This suggests that the base sequence and expression level of mRNAs may be related to the occurrence and severity of RP. In recent RNA-sequencing-based projects, we developed several computational prediction models integrated with RNA-Seq data that had potential for predicting the efficacy of targeted therapies for lung cancer and the diagnosis and prognosis of rectal cancer (7–9). Because mRNA is an important information carrier in the pathogenesis of RP, we integrated RNA-Seq data from individual patients into a prediction model and evaluated its predictive ability for the occurrence of RP in chest radiotherapy, to establish a reliable reference for optimizing the clinical efficacy of radiotherapy.

## Methods and Materials

### Study Design and Patients

**Figure 1:**
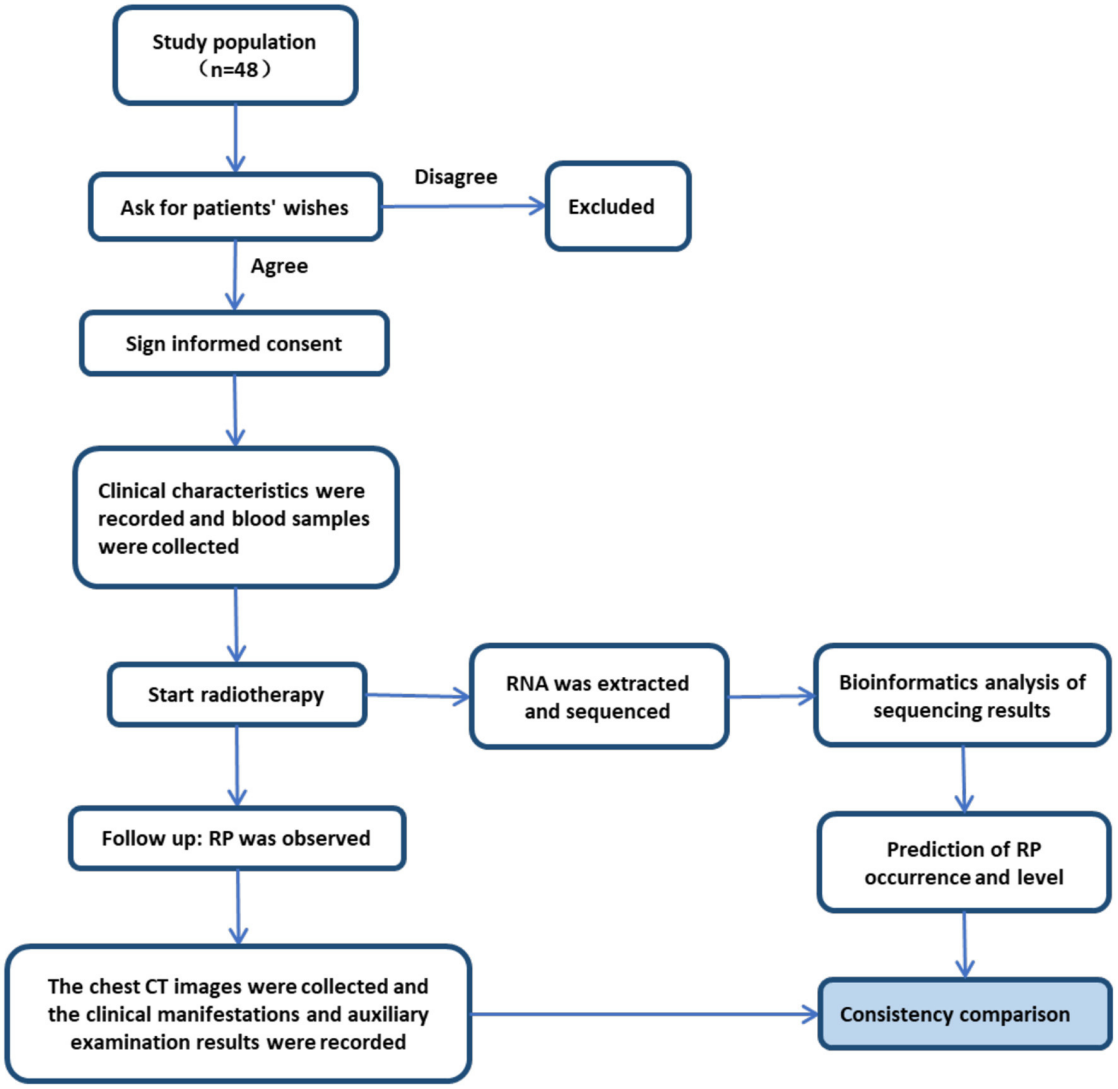
Study design workflow.

Patients who were diagnosed with lung cancer and treated with chest radiotherapy from October 2018 to December 2020 in Renmin Hospital of Wuhan University and the First Affiliated Hospital of Zhengzhou University were recruited for this study. The clinical information collected from the patients during treatment included sex, age, smoking history, history of chemotherapy, pathological type, tumor stage, target exposure dose, whole-lung-exposure dosage parameters (V5, V10, V20, and V30), and single-lung-exposure dosage parameters (V5, V10, V20, and V30). Clinical observation of the patients included clinical symptoms, lung imaging, and signs after radiotherapy. When RP occurred, the time from the beginning of radiotherapy to the diagnosis (days) was recorded, and the severity of RP was graded according to the RTOG diagnostic criteria (10). For each participant, a peripheral blood sample was collected before radiotherapy, and RNA sequencing of these blood samples was conducted. The RNA-Seq data were imported into a genome-scale molecular model for pathway flux analysis. Subsequently, an RP scoring system was established to predict the occurrence of RP for individual patients, and the results were compared with the clinically observed occurrence of RP during the course of radiotherapy [Figure 1; formula (1, 2)]. Written informed consent was obtained for all participants. This study was approved by the ethics committee of Renmin Hospital of Wuhan University.

### RNA Sequencing Study and Computational Prediction of RP Occurrence

For each participant, 10 ml of intravenous blood was collected 3–5 days prior to chest radiotherapy and stored in a blood RNA storage tube (TempusTMBlood RNA Tube) at −80°C. The collected blood samples were then thawed at room temperature (18–25°C) and the sample solution was transferred to a clean 50 ml tapered test tube. A total RNA extraction kit (TempusTMSpinRNA Isolation Kit) was used to extract the total RNA in the blood sample, following the manufacturer's instructions. The extracted RNA was then stored at −80°C. The globin mRNA was removed from the total RNA using the corresponding kit (GLOBINclearTMKit) following the manufacturer's instructions, and the isolated and purified RNA was then prepared for sequencing. The SMARTer Stranded Total RNA-Seq Kit v2 (Takara Bio Inc, Japan) was used to complete genomic cDNA synthesis, add connectors and indexes, use R-Probes to remove cDNA fragments from rRNA in the reverse transcription, delete interference from RNA from the library data in the sample, and finally expand the final RNA-Seq library. The captured RNA samples were then sequenced in a paired end manner using an Illumina NovaSeq 6000 platform. Afterward, the processed RNA-Seq data were imported into our computer prediction model [molecular signaling map, MSM (11)] for pathway flux calculation F(p).

(1) $$\text{F}\left(\text{p}\right)\text{=}\sum {\text{I}}_{\text{i}}\left(\text{reaction},\text{role}\right)\text{/N}\left(\text{P}\right))-\text{flux}\left(\text{Crosstalk}\left(\text{P}\right)\right)),$$

where N(P) is the number of reactions of the pathway P, and crosstalk(P) is the crosstalk pathways related to the pathway P. Through the flux comparative analysis (12), the significant differences (S) of pathway flux derived from the RNA-Seq data of each participant before (RP_control_) and after (RP_treat_) radiotherapy were calculated.

(2) $$\begin{array}{r} \text{S}=\text{lo}{\text{g}}_{2}\left(\text{F}\left(\text{R}{\text{P}}_{\text{treat}}\right)/\text{F}\left(\text{R}{\text{P}}_{\text{control}}\right)\right) \end{array}$$

A negative or zero value of S indicates a lower probability of RP occurrence, whereas a positive value indicates a higher probability of RP. The severity level of RP can be directly related to the value of S.

### Follow-Up Study and Assessment of RP Severity

After starting chest radiotherapy, clinical observations were continuously conducted to record the symptoms and signs of individual participants, including physical status score, oxygen saturation, breathing rate, pulse, and heart rate. For those who completed radiotherapy, chest CT and other related tests were carried out 1 and 3 months after the end of treatment, in strict accordance with the clinical treatment regulation. For patients resting at home, regular telephone follow-ups were performed. If patients experienced a cough, shortness of breath, a low fever, chest tightness and/or other symptoms, they were notified to carry out relevant examinations at any time. The occurrence of RP was determined through a chest CT image and the clinical symptoms and signs. The severity of RP was graded according to the RTOG standard classification.

### Statistical Analysis

A non-parametric Mann–Whitney *U*-test was applied to analyze the difference between the clinically observed RP occurrences and the predicted results of the RP scoring system. Univariant analysis (SPSS version 10.0) was applied to identify potential RP-related risk factors, including age, sex, smoking history, chemotherapy history, tumor stage, pathological type, and radiation exposure dose (full dose, V5, V10, V20, and V30). The identified potential risk factors were further analyzed by a multivariant approach in R (4.0.2). The prediction results were evaluated through ROC curve analysis between the clinical diagnostic group and the predicted RP score group.

## Results

A total of 48 lung cancer patients (median age, 62 years; age range, 35–80 years; 83.4% male) met the inclusion criteria and were included in this study. The demographic, clinical, and treatment data of this cohort are summarized in Table 1. Of these patients, 18 patients (37.5%) had small-cell lung cancer, 17 (35.4%) had lung squamous cancer, 11 (22.9%) had lung adenocarcinoma, and the remaining 2 (4.2%) had invasive breast cancer. Most patients were in disease stage II (20, 41.7%) and III (20, 41.7%), although five (10.4%) and three patients (6.3%) were in disease stages I and IV, respectively. Before radiotherapy, 44 patients (91.7%) received chemotherapy and 4 (8.3%) did not. In this cohort, 27 patients (56.3%) were smokers and 21 (43.8%) were non-smokers. The mean radiotherapy exposure dose in this cohort was 52.6 ± 11.8 Gy, the mean bilateral lung V5 was 42.75 ± 15.96%, the mean bilateral lung V20 was 17.34 ± 7.38%, the mean bilateral lung V10 was 30.10 ± 12.22%, and the mean bilateral lung V30 was 11.19 ± 5.50%.

**Table 1:** Relationship between clinical data and RP.

	**RP**	**Non-RP**	**χ^**2**^**	***P***
**Gender**			1.505	0.220
Male	22	19		
Female	2	5		
**Age (years old)**			2.231	0.693
<50	1	3		
50–59	7	6		
60–69	14	12		
70–79	2	2		
≥80	0	1		
**Smoking history**			0.762	0.383
Yes	15	12		
No	9	12		
**Chemotherapy**			0.000	1.000
Yes	22	22		
No	2	2		
**Pathological type**			2.385	0.303
Small cell lung cancer	8	10		
Adenocarcinoma	5	8		
Squamous cell carcinoma	11	6		
**Stage**			9.397	0.024
I	3	2		
II	8	12		
III	13	7		
IV	0	3		
**V5**			4.825	0.028
<37.17%	3	10		
≥37.17%	21	14		
**V10**			3.442	0.064
<26.33%	11	11		
≥26.33%	13	13		
**V20**			4.409	0.036
<15.11%	6	12		
≥15.11%	18	12		
**V30**			3.151	0.076
<9.62%	6	13		
≥9.62%	18	11		
**Radiation exposure dose**			8.021	0.005
<50.68 Gy	3	13		
≥50.68 Gy	21	11		

During this study, a clinical occurrence of RP of varying severity was observed in 24 patients (50.0%). Specifically, 17 cases of RP were evaluated as low grade (level 1 or 2), while seven cases of RP were evaluated as high grade (level 3 or 4). The average exposure dose in the target area of patients with an RP occurrence was 58.71 Gy, with the bilateral lung V5, V10, V20, and V30 being 47.04, 33.87, 19.90, and 12.76%, respectively. The average exposure dose in the target area of patients who did not experience RP was 50.68 Gy, with the bilateral lung V5, V10, V20, and V30 being 37.17, 26.33, 15.11, and 9.62%, respectively. The average exposure dose in the target area of patients with an RP level <2 was 50.68 Gy, with the bilateral lung V5, V10, V20, and V30 being 39.7, 27.50, 15.53, and 9.94%, respectively. The average exposure dose in the target area of patients with an RP level <3 was 51.77 Gy, with the bilateral lung V5, V10, V20, and V30 being 40.93, 28.38, 16.20, and 10.36%, respectively.

**Figure 2:**
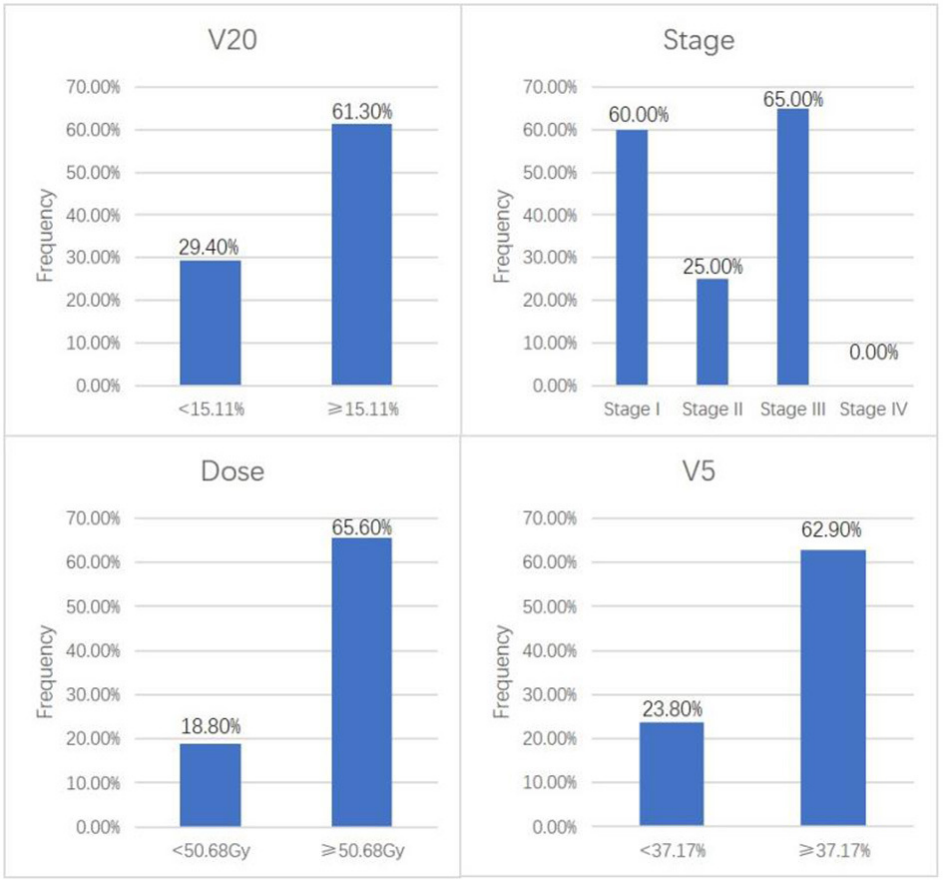
Univariate analysis showing the incidence of RP with statistically significant factors.

The univariant analysis results showed that an increased target exposure dose (*p* = 0.005), a later tumor stage (*p* = 0.024), and an increased bilateral lung V5 (*p* = 0.028) and V20 (*p* = 0.036) were significantly associated with the occurrence of RP (Figure 2; Table 1). An increased bilateral lung V5 (*p* = 0.000) and V20 (*p* = 0.003), and a history of smoking (*p* = 0.016) were significantly associated with the occurrence of RP at a level ≥ 2 (Table 2). Of note, patients with a bilateral lung V5 ≥ 39.70% or V20 ≥ 15.53%, or a history of smoking had a greater increase in risk for the occurrence of RP at a level ≥ 2 (Table 2).

**Table 2:** Relationship between clinical data and RP at a level ≥ 2.

	**≥2. level RP**	** <2. level RP**	**χ^**2**^**	***P***
**Gender**			0.680	0.410
Male	12	19		
Female	1	6		
**Age (years old)**			4.179	0.382
<50	0	4		
50–59	4	9		
60–69	9	17		
70–79	0	4		
≥80	0	1		
**Smoking history**			5.829	0.016
Yes	11	16		
No	2	19		
**Chemotherapy**			1.621	0.203
Yes	13	31		
No	0	4		
**Pathological type**			2.826	0.243
Small cell lung cancer	4	14		
Adenocarcinoma	2	11		
Squamous cell carcinoma	7	10		
**Stage**			6.317	0.097
I	1	4		
II	2	18		
III	8	11		
IV	2	2		
**V5**			12.306	0.000
<39.70%	2	15		
≥39.70%	11	20		
**V10**			3.720	0.054
<22.50%	2	16		
≥22.50%	11	19		
**V20**			8.698	0.003
<15.53%	1	18		
≥15.53%	12	16		
**V30**			0.579	0.447
<9.94%	4	15		
≥9.94%	9	20		
**Radiation exposure dose**			3.227	0.072
<50.68 Gy	2	15		
≥50.68 Gy	11	20		

**Figure 3:**
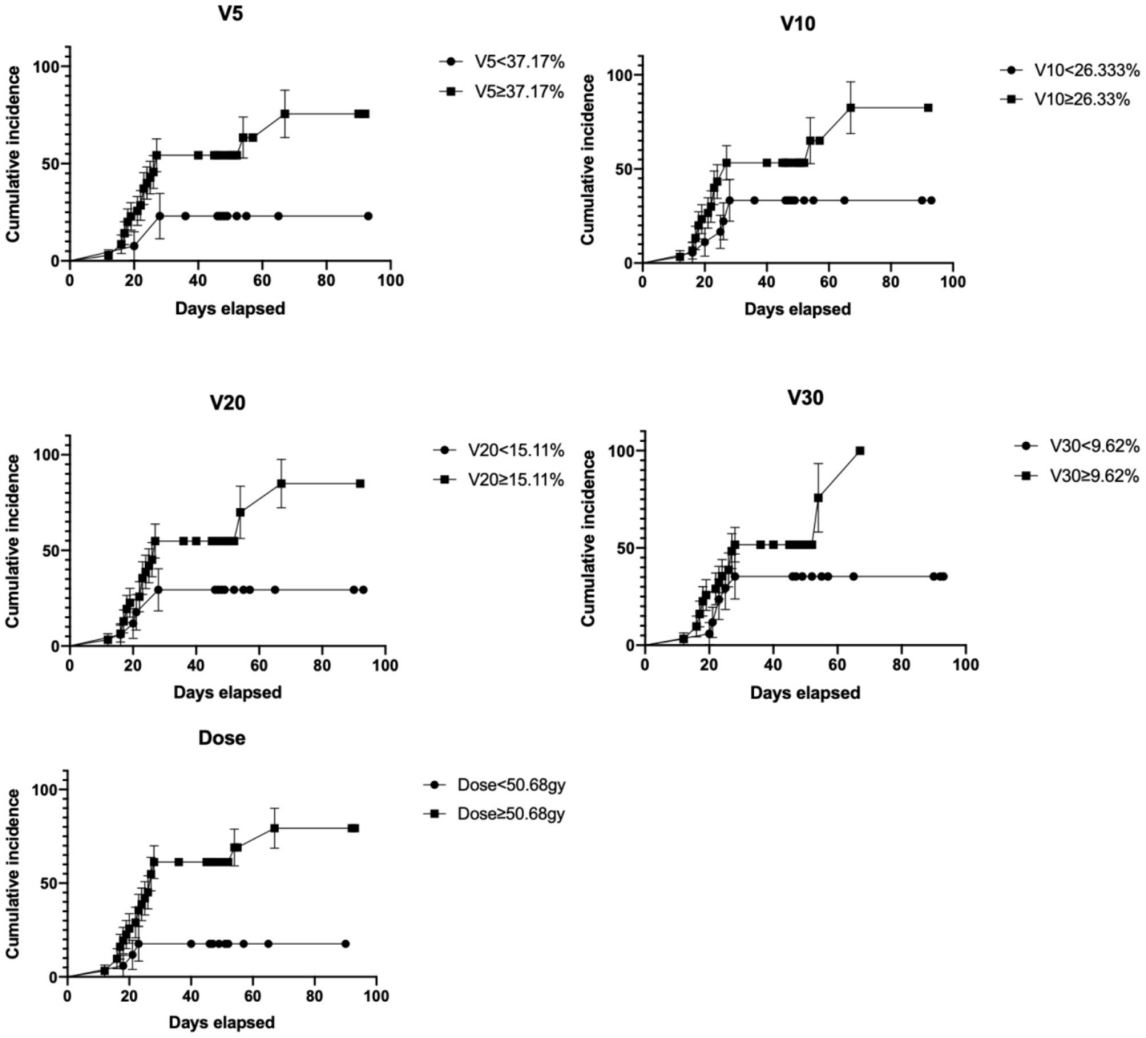
Cumulative incidence rate curves of RP under different dosimetric parameters. The map shows the cumulative incidence rate of RP in the observation period (2–10 w) under different physical parameters (V5, V10, V20, V30, and total target dose).

A history of smoking (*p* = 0.012), a later tumor stage (*p* = 0.009), and an increased bilateral lung V5 (*p* = 0.027) and V20 (*p* = 0.016) were significantly associated with the occurrence of RP at a level ≥ 3 (Table 3). Of note, patients with a bilateral lung V5 ≥ 40.93%, V20 ≥ 16.20%, tumor stage III or later, or a history of smoking faced a significantly increased risk for the occurrence of RP at a level ≥ 3 (Table 3). However, the radiation exposure doses, including the single-lung V5, V10, V20, and V30, were all shown to be unrelated to the occurrence of RP. The Cox proportional-hazards model analyzed all the significant risk factors obtained by the univariant analysis, and the results showed that the radiation exposure dose of the bilateral lung V5 was an independent risk factor for the occurrence of RP (*p* <0.05), while the bilateral lung V10 was an independent risk factor for the occurrence of RP at a level ≥ 2 (*p* <0.05). The cumulative incidence rates related to different radiation exposure parameters are shown in Figure 3, indicating the incidence rates of the occurrence of RP within the 3-month period of clinical observation under different exposure doses. The curves of the cumulative incidence rate of different dosimetric parameters, as shown in Figure 3, suggest that the frequency of RP occurs within a certain time of observation (3 months) under different dosimetric parameters.

**Table 3:** Relationship between clinical data and RP at a level ≥ 3.

	**≥3. level RP**	** <3. level RP**	**χ^**2**^**	***P***
**Gender**			1.399	0.237
Male	7	34		
Female	0	7		
**Age (years old)**			1.994	0.737
<50	0	4		
50–59	2	11		
60–69	5	21		
70–79	0	4		
≥80	0	1		
**Smoking history**			6.374	0.012
Yes	7	20		
No	0	21		
**Chemotherapy**			0.745	0.388
Yes	7	37		
No	0	4		
**Pathological type**			0.686	0.709
Small cell lung cancer	3	15		
Adenocarcinoma	1	12		
Squamous cell carcinoma	3	14		
**Stage**			12.473	0.009
I	0	5		
II	0	20		
III	7	13		
IV	0	3		
**V5**			4.917	0.027
<40.93%	0	18		
≥40.93%	7	23		
**V10**			2.764	0.079
<28.38%	1	18		
≥28.38%	6	23		
**V20**			5.854	0.016
<16.20%	0	20		
≥16.20%	7	21		
**V30**			2.891	0.089
<10.36%	1	20		
≥10.36%	6	21		
**Radiation exposure dose**			1.600	0.206
<51.77 Gy	1	16		
≥51.77 Gy	6	25		

**Figure 4:**
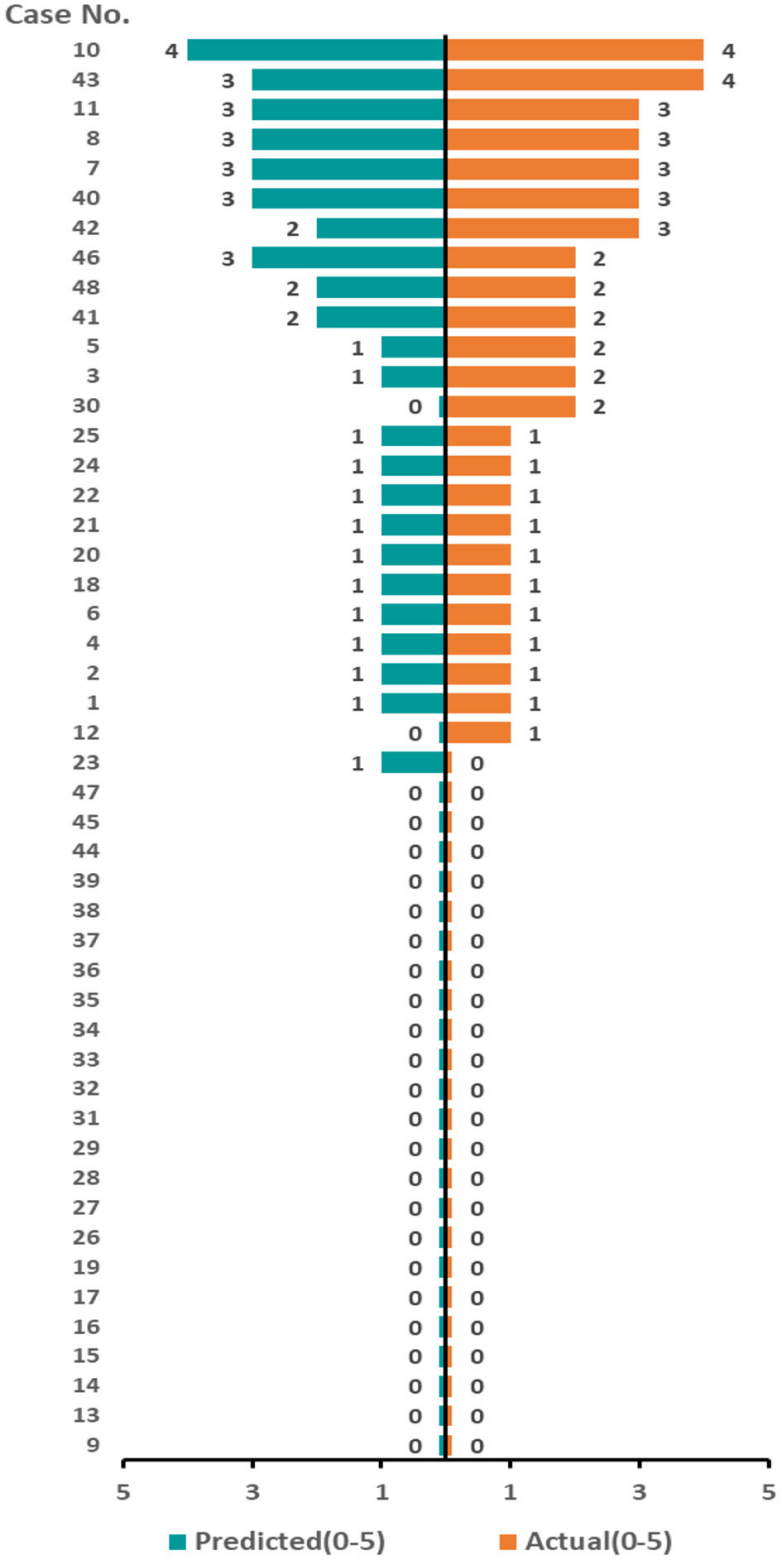
Predicted value for RP compared with the clinical observations according to the RNA sequencing. Each row represents a patient. The predicted results (orange band) correspond to the clinical observation results (blue band) row by row. The length of the band is related to the corresponding RP level. The scores of the predicted or actual observation results are symbolized by the length of each band. The higher the score, the longer the band, indicating a higher level of predicted or observed RP, respectively.

**Figure 5:**
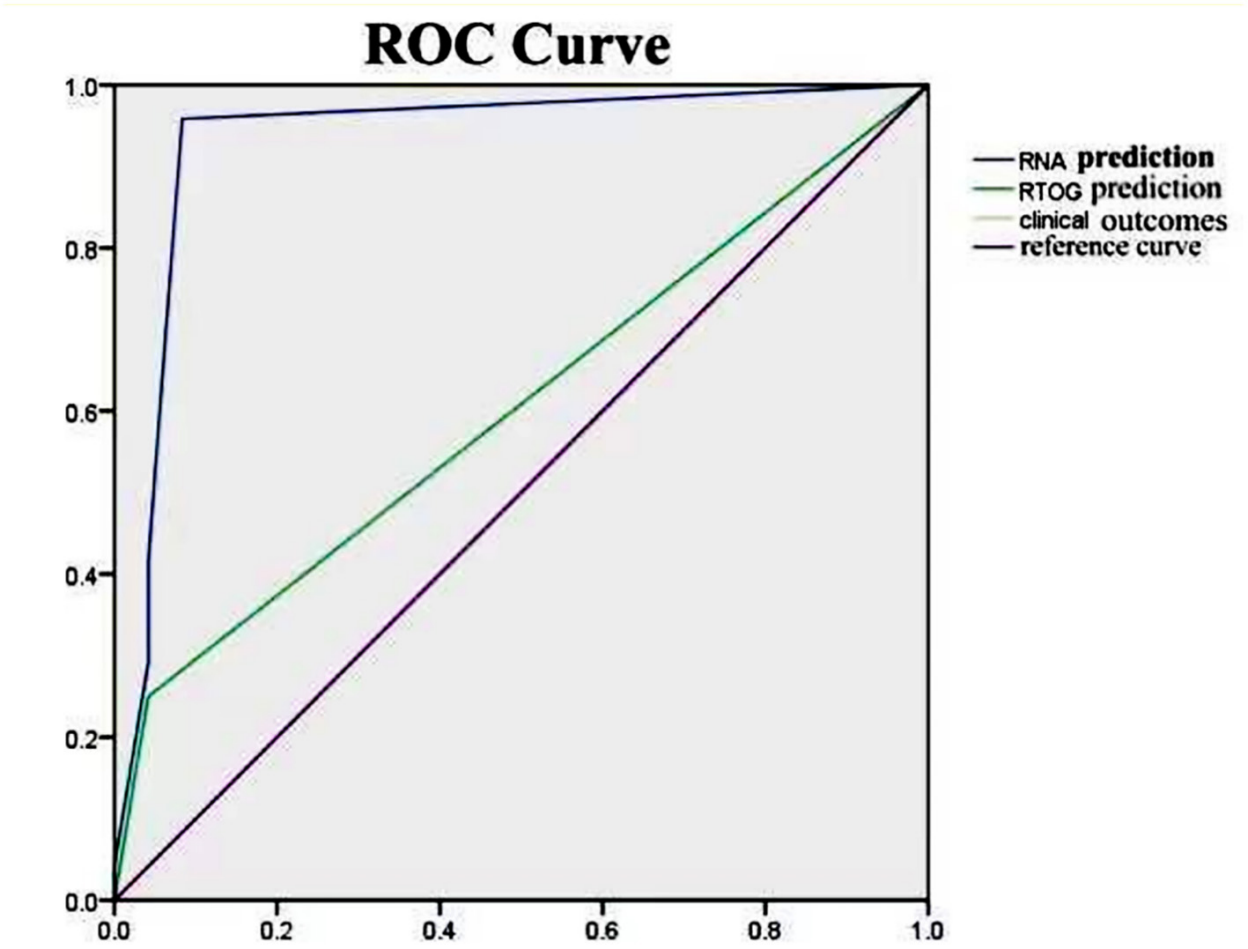
ROC curve of RP prediction results and clinical observation results based on the RNA sequencing and RTOG physical parameters. The chart compares the prediction results among the RILI, RTOG, and clinically observed RP outcomes. The prediction results of RILI and RTOG have AUC of 0.933 and 0.604, respectively. This indicates that the prediction results of RILI are significantly better than those of RTOG.

From peripheral blood samples in this cohort, RNA-Seq libraries were successfully extracted and checked with the quality control procedure stated in the Methods section. Then, RNA-Seq data from each patient were integrated into the computational prediction model, MSM. Subsequently, genome-scale pathway flux analysis was performed to generate S values (as detailed in the Methods section). Afterward, each S value was compared to the clinically observed outcome of the corresponding patient, as shown in Figure 4. A Spearman rank sum test showed a positive significant correlation between both groups (rho = 0.915; *p* <0.0001), with an overall consistency of 81.25% (39/48; 95% CI = 77.91–99.15%). The median of the S values was 0.537 (range, 0.0–1.2). In this cohort, 8 (16.7%) and 17 (35.4%) patients were predicted with a high probability to experience RP at a level ≥ 3 and ≤ 2, respectively, during radiotherapy. The remaining 23 patients (47.9%) were predicted to receive radiotherapy without experiencing RP. The non-parametric rank sum test indicated no significant difference between the predicted results from the RP scoring system and the clinically observed occurrence of RP in this cohort (*p* = 0.405), regardless of the total incidence of RP or the occurrence data of the different levels of RP. Moreover, ROC analysis was performed to compare the predictions of the RP scoring system with those of the classical RTOG radiation physical parameters (Figure 5). The comparison result showed that the area under the curve (AUC) of the prediction from the RP scoring system and the ROTG classification were 0.933 (95% CI = 0.89–1.00) and 0.604 (95% CI = 0.44–0.77), respectively. This strongly indicated that the predictive ability of the RP scoring system was superior to that of the ROTG classification for the diagnosis of RP occurrence in this cohort, with a significantly higher sensitivity and specificity.

## Discussion

Generally speaking, the efficacy of radiotherapy for most tumors is positively correlated with the dose of irradiation (13). The general principle for developing radiotherapy strategies is to maximize the dose to tumor-affected tissue while protecting healthy tissue (14). However, under the guidance of evidence-based medicine, the dose received by most patients is determined by the best irradiation dose for the group of patients. Individualized determination of the optimal dose for radiotherapy is a highly challenging task, because slight change of dose for individuals may directly lead to damage of healthy tissues or dramatical reduction of therapeutic efficacy during the course of radiotherapy (15–17). Thus, precise screening of patients with a high risk of developing RP before radiotherapy becomes an essential and important prerequisite for individualized radiotherapy.

Currently, the predictive biomarkers commonly used in a clinical prognosis for RP include clinical phenotypes (including sex, age, smoking history, chemotherapy history, types of tumor pathology, and clinical stages), biological biomarkers (such as levels of IL-6, IL-8, TGF-β, and other cytokines in the serum), and radiophysical properties (including V5, V20, V10, V30, and mean lung dose). Nevertheless, the predictive ability of these biomarkers varies greatly between studies. This suggests that it may be insufficient to only apply information related to clinical phenotypes and/or tumor characteristics of individual patients for the prognosis of RP during radiotherapy. Furthermore, the development of RP is a dynamic process, involving multiple stages and factors, including tissues, cells, and molecules (18, 19). The influence of radiomics, biomics, and clinically relevant risks also needs to be considered comprehensively to accurately assess the risk of developing RP for patients receiving radiotherapy, and to achieve the final goal of individualized treatment.

Wide use of omics data and machine learning has largely promoted advances in medicine and drug development, as well as providing new opportunities. In the present study, we used a genome-scale flux analysis approach integrating a molecular signaling map based on patient-individualized RNA sequencing data. Previous studies have demonstrated that this approach, based on transcriptomic data, was able to quickly predict the efficacy of different treatments received by patients in ~1 h (20). Compared with observational results, a computer assisted prediction approach can be much easier, more precise, and have a higher efficacy than traditional strategies. To determine the predictive efficacy of this computational molecular model for the development of RP in patients receiving radiotherapy, we compared the predicted outcomes before radiotherapy of 48 patients with the observed clinical outcomes of these patients after radiotherapy. The results of our comparison showed that there was extremely high consistency (81.25%) between the predicted results and the observational outcomes, and ROC analysis suggested that our computational prediction provides a higher accuracy (AUC, 0.933; range, 0.910–0.958; *p* <0.001) for the prediction of RP compared with other biomarkers such as clinical phenotypes.

Our study also determined the predictive efficacy of common clinical phenotypes for RP. We evaluated the associations between the development of RP and sex, age, smoking history, chemotherapy history, pathological type, irradiation dose of target area, tumor stage, and the V5, V10, V20, and V30 values of the bilateral lung and the affected lung tissue. The results of the univariate analysis indicated that the irradiation dose of the target area, the tumor stage, and the bilateral V5 and V20 values were significantly associated with the development of RP (*p* <0.05) (see Table 1). In contrast, the V5, V10, V20, and V30 values of the affected lung tissue were not correlated with the development of RP. A Cox multivariate analysis showed that the bilateral V5 value was an independent risk factor for the development of RP, while the V20 value was an independent risk factor for the development of RP at grade 2 and above (*p* <0.05). Consistent with our results, Bryan et al. (21) observed that the V20 value and the mean lung dose were the main factors affecting the incidence of RP from a study including 281 patients with non-small cell lung cancer. The incidence of RP increased from 4.3 to 16.4% when V20 ≥ 4.3%. Moreover, Kong et al. (22) found that the incidence of RP at grade 2 and above was 16% when V20 ≤ 27%, while the incidence increased to 48% when V20 > 27%.

The present study had several limitations. First, a statistical bias may exist given the small sample size of this cohort, limited follow-up data, and missing patient data. Second, some of our results contradict previous reports, for instance, the V20 threshold was predictive for the development of RP at grade 2 or higher, possibly due to the small sample size and the different clinical stages of the patients recruited. Third, the predictive model in our study was determined according to the results of molecular signaling maps (MSM), which has demonstrated its potential in predicting the efficacy of targeted therapy in patient-derived xenograft models. However, RP is a pathophysiological process involving multiple factors, including the immune response, the release of substances after tumor necrosis, the status of lung tissue, and radiophysical properties. Future studies are warranted to further investigate whether it remains the best strategy to predict the risk of developing RP based on transcriptomic data alone, or whether more comprehensive and accurate predictive models for RP can be established by utilization of other high-throughput data, such as metabolomics, clinical parameters, or even with single-cell data.

In conclusion, we performed an initial exploration of the utility of a computer-assisted predictive model to predict the development of RP. Our study demonstrated that the computer-assisted predictive model based on RNA sequencing data was able to precisely predict the risk of developing RP in patients undergoing thoracic radiotherapy. Our strategy showed a significantly higher predictive efficacy compared with traditional strategies, providing a useful tool to precisely predict the risk of RP and thus promoting the development of individualized radiotherapy.

## Data Availability Statement

The data that support the findings of this study are available on request from the corresponding author.

## Ethics Statement

The studies involving human participants were reviewed and approved by the ethics committee of Wuhan Renmin Hospital. The patients/participants provided their written informed consent to participate in this study.

## Author Contributions

SY, YY, YD, YL, LY, YH, YG, and QL: acquisition, analysis, or interpretation of data. SY, YY, YD, JLiu, and QS: drafting of the manuscript and conception design. SY, YY, JY, and JLi: statistical and bioinformatical analysis. SY, YL, DY, HG, BX, and JLi: critical revision of the manuscript for important intellectual content. JLi and QS: supervision. All authors contributed to the article and approved the submitted version.

## Conflict of Interest

The authors declare that the research was conducted in the absence of any commercial or financial relationships that could be construed as a potential conflict of interest.

## Publisher's Note

All claims expressed in this article are solely those of the authors and do not necessarily represent those of their affiliated organizations, or those of the publisher, the editors and the reviewers. Any product that may be evaluated in this article, or claim that may be made by its manufacturer, is not guaranteed or endorsed by the publisher.
